# Leclercia adecarboxylata-Associated Catheter-Related Bloodstream Infection in a Hemodialysis Patient: A Case Report and Literature Review

**DOI:** 10.7759/cureus.112326

**Published:** 2026-07-09

**Authors:** Surajit Chakraborty, Jai Ranjan, Nabadwip Pathak, Saurabh Nayak, Bhawna Sharma

**Affiliations:** 1 Microbiology, All India Institute of Medical Sciences, Bathinda, Bathinda, IND; 2 Nephrology, All India Institute of Medical Sciences, Bathinda, Bathinda, IND

**Keywords:** catheter-related bloodstream infection, chronic kidney disease, gentamicin lock therapy, hemodialysis, leclercia adecarboxylata

## Abstract

*Leclercia adecarboxylata* (*L. adecarboxylata*) is a motile, gram-negative bacillus from the Enterobacteriaceae family, traditionally considered a low-virulence environmental organism and gut commensal. However, increasing reports have pointed to its emerging role as an opportunistic pathogen capable of causing clinically significant infections in both immunocompromised and immunocompetent individuals. We describe a case of catheter-related bloodstream infection (CRBSI) in a 49-year-old female with end-stage renal disease on maintenance hemodialysis via a tunneled central venous catheter. She presented with persistent fever, myalgia, and elevated inflammatory markers. Paired blood cultures from the catheter and peripheral vein flagged positive within 24 hours, with earlier positivity in the catheter sample, suggesting CRBSI. Pure growth of gram-negative bacilli was identified as *L. adecarboxylata* using VITEK®2 and later re-confirmed by matrix-assisted laser desorption/ionization time-of-flight mass spectrometry (MALDI-TOF MS). Antimicrobial susceptibility testing demonstrated broad susceptibility except to select cephalosporins. The patient was successfully treated with intravenous meropenem and gentamicin lock therapy, resulting in complete clinical resolution. This case highlights the emerging clinical importance of *L. adecarboxylata* as a cause of CRBSI in hemodialysis patients. Accurate identification and prompt, targeted treatment are crucial for the best outcomes. It also underscores the organism’s ability to colonize devices and the need for increased clinical vigilance. Given the rise in reports of antimicrobial resistance, continuous surveillance and further research are necessary to better understand its pathogenic potential and to develop optimal management strategies.

## Introduction

*Leclercia adecarboxylata* (*L. adecarboxylata*) is a motile, Gram-negative bacillus belonging to the Enterobacteriaceae family. The organism was initially classified as "enteric group 41" and subsequently described as *Escherichia adecarboxylata* by Henri Leclerc in 1962 [1]. Based on distinctive biochemical characteristics (most notably its inability to decarboxylate lysine, in contrast to* Escherichia coli*, which underlies the species epithet *adecarboxylata*) as well as taxonomic differences, the organism was reclassified in 1986 into a newly established genus, *Leclercia*, with *L. adecarboxylata* as the sole species. The genus name is eponymous, honoring Henri Leclerc for his original identification of the bacterium [1,2].

*L. adecarboxylata* is widely distributed in aquatic and terrestrial environments and constitutes part of the normal intestinal microbiota of mammals, including humans [3]. Since its initial description, reports of human infections attributed to this organism have been relatively infrequent and limited, leading to its perception as a low-virulence opportunistic pathogen. However, an increasing number of cases have been documented in recent years, demonstrating its capacity to cause monomicrobial infections in both immunocompromised and immunocompetent hosts [1,4-6]. Consequently, *L. adecarboxylata* is now being increasingly recognized as an emerging pathogen of clinical relevance.

Here, we report a case of catheter-related bloodstream infection (CRBSI) caused by *L. adecarboxylata* in a hemodialysis patient with a central venous tunneled hemodialysis catheter in situ.

## Case presentation

A 49-year-old female patient with a known history of chronic kidney disease (CKD) receiving regular maintenance hemodialysis through a central venous tunneled hemodialysis catheter at a local facility in her hometown was brought to the nephrology outpatient department (OPD) of our tertiary care centre. Upon her visit to the OPD, she complained of persistent moderate- to high-grade fever for the last 10 days associated with generalized myalgia, nausea, vomiting, and continuous fatigue. On examination, her blood pressure was 140/95 mmHg, pulse rate 90/min, and body weight 54.5 kg. Laboratory investigations of the patient are detailed in Table 1. 

**Table 1:** Results of laboratory investigations at initial presentation

Parameters	Patient values	Reference range
Hematological parameters		
Total leukocyte count (TLC)	11.06 × 10³/µL	4.00-11.00 × 10³/µL
Neutrophil count	9.45 × 10³/µL	2.00-7.00 × 10³/µL
Red blood cell (RBC) count	2.11 × 10⁶/µL	3.50-5.50 × 10⁶/µL
Hemoglobin	7.1 g/dL	11.0-16.0 g/dL
Platelet count	191 × 10³/µL	150-450 × 10³/µL
Biochemical parameters		
Urea	123.9 mg/dL	15-45 mg/dL
Creatinine	9.51 mg/dL	0.6-1.10 mg/dL
Inflammatory markers		
C-reactive protein	3.44 mg/L	<6 mg/L

**Figure 1:**
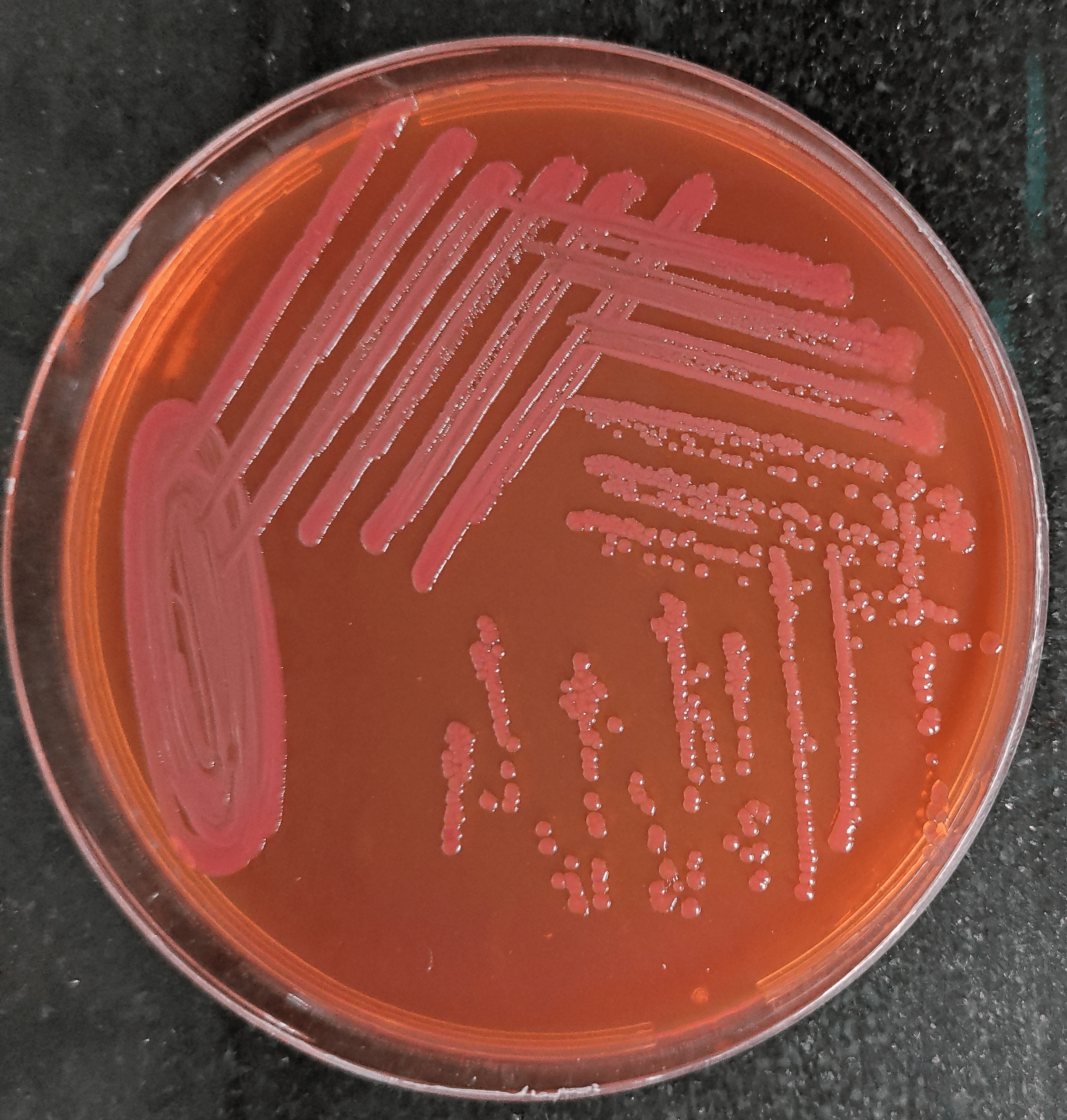
Leclercia adecarboxylata lactose-fermenting colonies on MacConkey agar

Paired blood samples obtained from both the central venous catheter and a peripheral vein were submitted to the Department of Microbiology for culture using the BACT/ALERT 3D automated blood culture system (bioMérieux, Marcy l'Etoile, France). Both cultures flagged positive within 24 hours. The blood culture from the central line catheter was positive approximately four hours before the peripheral venous blood. Both the flagged positive blood cultures were subsequently sub-cultured onto sheep blood agar and MacConkey agar plates. Gram staining of the direct smear from the positive blood culture bottles revealed gram-negative bacilli. After overnight incubation at 37 °C, pure growth of greyish-white colonies was observed on blood agar, while pale pink, lactose-fermenting colonies were noted on MacConkey agar (Figure 1).

Both the colonies were catalase positive and oxidase negative. The isolates from both agar media were identified as *Leclercia adecarboxylata *by a VITEK®2 COMPACT system with 98% probability using a GN card (bioMérieux), and antimicrobial susceptibility was obtained with an N-405 card (Table 2).

**Table 2:** Antimicrobial susceptibility profile of the L. adecarboxylata isolate from the present case (as reported to guide clinical management) S: susceptible; I: intermediate; R: resistant As per CLSI M100, 35th ed., 2025 [7].

Antimicrobial agents	Zone of Inhibition (ZoI) size (mm)	MIC (mg/mL)	Interpretation
Piperacillin/tazobactam	-	≤4	S
Cefuroxime	-	>64	R
Cefuroxime axetil	-	>64	R
Ceftriaxone	-	>64	R
Cefoperazone/sulbactam	-	≤8	S
Cefepime	-	16	R
Ertapenem	-	≤0.12	S
Imipenem	-	≤0.25	S
Meropenem	-	≤0.25	S
Amikacin	-	2	S
Gentamicin	-	≤1	S
Ciprofloxacin	-	0.25	S
Colistin	-	≤1	I
Trimethoprim/sulfamethoxazole	-	≤20	S
Chloramphenicol	30	-	S
Levofloxacin	24	-	S

A simultaneous manual AST was conducted with other recommended methods (Kirby-Bauer disk diffusion, Colistin agar test, and Colistin broth disk elution), and results were interpreted as per the CLSI 2025 guidelines [7], which correlated with susceptibility obtained through the automated method. Prior to the initiation of antimicrobial therapy, a second set of paired blood samples obtained from both the central venous catheter and a peripheral vein was also collected and processed as previously described.

Both the blood cultures from the second set were also positive, and *L. adecarboxylata* with the same susceptibility pattern was again isolated from both samples. The identity of the pathogen was further confirmed using matrix-assisted laser desorption/ionization time-of-flight mass spectrometry (MALDI-TOF MS) (bioMérieux), with a confidence value of 99.9.

Based on the antimicrobial susceptibility profile, the patient was treated with intravenous meropenem (500 mg once daily for 15 days) in combination with gentamicin lock therapy (1 mg gentamicin with 1,000 IU unfractionated heparin per mL). Following therapy, the infection resolved, and the patient became afebrile.

## Discussion

*L. adecarboxylata* has been increasingly recognized as a clinically relevant pathogen, having been isolated from a broad spectrum of infectious conditions in both immunocompromised and immunocompetent hosts. The organism has been reported to cause infections either as a sole pathogen or as part of polymicrobial etiology. Documented clinical manifestations include colonic perforation, peritonitis, wet gangrene, soft tissue and surgical site infections, septic arthritis, pneumonia, and bacteremia, among others [1,4,8]. More recently, monomicrobial spinal and neurosurgical infections attributed to *L. adecarboxylata* have been described, further expanding the recognized clinical spectrum of this organism [5,6]. However, although *L. adecarboxylata* is increasingly recognized as a causative agent across a spectrum of infections, fatal outcomes have been reported only rarely [4]. To date, no definitive association has been established between monomicrobial infection due to *L. adecarboxylata* and mortality.

Although bloodstream, urinary tract, and soft tissue infections constitute the most frequently reported presentations, device-associated infections due to *L. adecarboxylata* remain uncommon and under-reported. In the present case, the blood culture obtained from the central venous catheter flagged positive approximately four hours earlier than the peripheral venous sample, suggesting a probable CRBSI. *L. adecarboxylata* was isolated from both blood culture specimens.

*L. adecarboxylata* is primarily an environmental bacterium that also forms part of the normal intestinal microbiota of mammals [3]. In general, *L. adecarboxylata* isolates remain susceptible to a wide range of antimicrobial agents; however, emerging reports of multidrug-resistant strains are a cause for concern. Studies have identified multiple antimicrobial resistance mechanisms in *L. adecarboxylata*, including intrinsic resistance (e.g., to fosfomycin), as well as chromosomal and plasmid-mediated determinants associated with diverse mobile genetic elements, consistent with its environmental origin. These findings suggest active intra- and interspecies exchange of resistance genes. Notably, widespread β-lactam resistance has been attributed to plasmid-encoded extended-spectrum β-lactamases (ESBLs), particularly *bla*TEM-1 and *bla*CTX-M variants, followed by class-1 integron-integrase genes (*intl*1 genes) associated with resistance to folate pathway inhibitors [9,10]. There are also sporadic reports of aminoglycoside resistance and of carbapenemase (plasmid encoding *bla*IMP) producing *L. adecarboxylata* [3,10,11]. In contrast, the isolate in the present case demonstrated broad antimicrobial susceptibility (Table 2), despite its resistance to the third- (cefuroxime, ceftriaxone) and fourth-generation (cefepime) cephalosporins. The patient responded favorably to targeted systemic therapy with intravenous meropenem in combination with gentamicin antibiotic lock therapy. This combined approach resulted in clinical resolution of infection and defervescence, underscoring the importance of early identification and appropriate antimicrobial management in device-associated infections.

Accurate identification of *L. adecarboxylata* remains a diagnostic challenge due to its close metabolic and biochemical resemblance to *Escherichia coli*, which may lead to misidentification when conventional phenotypic methods are used [12]. This diagnostic limitation may partly explain the relatively small number of reported cases in the literature. In the current study, the organism was initially identified using the VITEK®2 COMPACT system, with definitive confirmation achieved by MALDI-TOF MS, highlighting the value of advanced diagnostic modalities in recognizing uncommon pathogens.

The repeated isolation of *L. adecarboxylata* from paired peripheral and catheter-drawn blood cultures obtained prior to initiation of antimicrobial therapy strongly supports its etiological role in catheter-related bloodstream infection rather than contamination. This case therefore adds to the limited but growing body of evidence documenting *L. adecarboxylata* as a cause of CRBSI in patients undergoing maintenance hemodialysis.

Patients with chronic kidney disease on hemodialysis and undergoing chemotherapy are inherently predisposed to bloodstream infections owing to immune dysregulation, frequent healthcare exposure, and the presence of indwelling intravascular devices [13,14]. CRBSIs in such patients with long-term intravascular devices are most often attributed to skin commensals, particularly *Staphylococcus epidermidis* and *Staphylococcus aureus*, and significantly less frequently to others [15]. Monomicrobial involvement of *L. adecarboxylata* in blood-stream infections, including CRBSIs, is rarely reported. Nevertheless, a small number of reports of polymicrobial etiology of bloodstream infections involving* L. adecarboxylata *exist, involving *Escherichia hermannii*, *Enterococcus faecalis*, *Pantoea agglomerans*, *Acinetobacter baumannii*, *Enterobacter cloacae *complex, etc. [15]

A limited number of case reports from different geographical regions have documented its involvement in catheter-related bacteremia, including CRBSI. These reports indicate that the organism has the ability to colonize and infect intravascular devices, particularly in susceptible hosts, such as patients undergoing hemodialysis, chemotherapy, etc. The details of published reports on *L. adecarboxylata* bacteremia linked to intravascular catheters are listed in Table 3.

**Table 3:** Published reports of L. adecarboxylata bacteremia linked to intravascular catheters ESRD, end-stage renal disease; CVC, central venous catheter; PICC, peripherally inserted central catheter; CKD, chronic kidney disease. ^#^MDR: Non-susceptibility to at least one antimicrobial agent in three or more distinct antimicrobial classes. ^$^Resistant to ampicillin, piperacillin, cephalothin, cefazolin, cefaclor, cefixime, cefotaxime, cefoperazone, aztreonam, amikacin, gentamicin, tobramycin, and trimethoprim-sulfamethoxazole.

Patient	Case	Year	Catheter	Antibiogram	Antibiotic treatment	Catheter management	Patient outcome	Ref.
81-year-old male	Hemodialysis (ESRD)	2009	Tunneled CVC in the subclavian vein	Susceptible to all tested antibiotics	A 15-day course of ceftriaxone combined with catheter lock therapy with ciprofloxacin	Catheter maintained	Infection clearance and recovery	[16]
72-year-old male	Hemodialysis (allograft rejection in renal failure)	2009	Tunneled CVC in the subclavian vein	Susceptible to all tested antibiotics	A 15-day course of meropenem combined with catheter lock therapy with gentamicin	Catheter maintained	Infection clearance and recovery	[16]
58-year-old male	Hemodialysis (ESRD)	2011	Tunneled CVC in internal jugular vein	Susceptible to all tested antibiotics	2 weeks of antibiotics (antibiotics not specified)	Catheter removed	Infection clearance and recovery	[17]
55-year-old male	Trauma	2012	Temporary CVC in subclavian vein	Susceptible to all tested antibiotics	A 10-day course of cefazolin	Catheter removed and replaced	Infection clearance and recovery	[18]
47-year-old female	On active chemotherapy for breast cancer	2012	PICC	MDR^#, $^	Cefepime (duration not specified)	Not specified	Infection clearance and recovery	[9]
81-year-old male	Hemodialysis (ESRD)	2013	Tunneled CVC in internal jugular vein	Susceptible to all tested antibiotics	1 week of gentamicin, 3 weeks of amoxicillin-clavulanic acid combined with catheter lock therapy with gentamicin	Catheter maintained	Infection clearance and recovery	[19]
48-year-old female	Peritoneal dialysis (ESRD)	2019	Peritoneal dialysis catheter	Susceptible to all tested antibiotics	A 21-day course of cefazolin	Catheter maintained	Infection clearance and recovery	[12]
50-year-old female	Hemodialysis (ESRD)	2020	Tunneled CVC in internal jugular vein	Susceptible to most of the tested antibiotics (except ampicillin, amoxicillin/clavulanate, piperacillin/tazobactam, ceftriaxone)	2 weeks of meropenem and gentamicin	Not specified	Infection clearance and recovery	[20]
34-year-old male	On active chemotherapy for metastatic sarcoma	2022	Tunneled CVC in internal jugular vein	Susceptible to ampicillin, levofloxacin, and Trimethoprim/Sulfamethoxazole	2 weeks of levofloxacin	Catheter removed	Infection clearance and recovery	[21]
38-year-old transgender female	Total parenteral nutrition (gastric and duodenal diffuse large B-cell lymphoma)	2023	Tunneled CVC	Susceptible to most of the tested antibiotics (except amoxicillin/clavulanic acid, fosfomycin, and trimethoprim/sulfamethoxazole)	2 weeks of piperacillin/tazobactam	Catheter removed and replaced with PICC	Infection clearance and recovery	[22]
54-year-old female	Active on chemotherapy for breast cancer	2026	Central vein- chemoport	Susceptible to all tested antimicrobial agents	Ciprofloxacin (500mg for 2 weeks)	Removed	Infection clearance and recovery	[15]

## Conclusions

The present case, in conjunction with previously reported instances, underscores the emerging role of *L. adecarboxylata* as a potential nosocomial pathogen, particularly in infections associated with temporary or permanent vascular access. As this organism can closely resemble *Escherichia coli*, accurate identification requires advanced diagnostic modalities such as MALDI-TOF MS or molecular diagnostic techniques. There have been reports of emerging antimicrobial resistance in this organism through multiple mechanisms. These observations highlight the need for heightened clinical awareness, accurate microbiological identification, and robust epidemiological surveillance to guide preventive and therapeutic strategies aimed at reducing device-associated infections in this vulnerable population.
